# Effects of Human Trampling on Soil Microbial Community Assembly in Yangzhou Urban Forest Park

**DOI:** 10.3390/microorganisms13122648

**Published:** 2025-11-21

**Authors:** Jingwei Lian, Liwen Li, Xin Wan, Dongmei He, Yingzhou Tang, Wei Xing, Yingdan Yuan

**Affiliations:** 1 Jiangsu Academy of Forestry, Nanjing 211153, China; 2 Jiangsu Yangzhou Urban Forest Ecosystem National Observation and Research Station, Yangzhou 225006, China; 3 College of Horticulture and Landscape Architecture, Yangzhou University, Yangzhou 225009, China

**Keywords:** foot-traffic disturbance, urban forest, soil physical, chemical properties, neutral community model, null model

## Abstract

Human trampling in urban forest parks has received increasing attention, yet its effects on microbial community assembly remain elusive. This study investigated how trampling influences soil physicochemical properties and microbial communities in Zhuyuwan Scenic Area. Neutral and null community models were used to analyze the effects of trampling on microbial assembly processes. Trampling altered both soil physicochemical properties and microbial diversity. Fungal richness differed significantly between control and light-trampling plots. Soil bulk density (SD) was strongly negatively correlated with other soil physical properties, which were positively intercorrelated. Model analyses showed that light trampling strengthened stochastic processes in bacterial community assembly, whereas heavy trampling reduced this effect. Increasing trampling intensity intensified the influence of stochastic processes on fungal community assembly. Bacterial communities were mainly shaped by heterogeneous selection, while fungal communities were primarily governed by dispersal limitation. These results enhance understanding of how trampling disturbance influences microbial community assembly and provide a theoretical basis for the ecological management and restoration of urban forest parks.

## 1. Introduction

Urban forest parks are public green spaces centered on forest landscapes, integrating natural ecology, cultural features, and recreational functions for citizens to engage in sightseeing, exercise, scientific education, and ecological conservation. They play a vital role in urban ecosystems by protecting biodiversity, purifying the environment, and providing residents with spaces for leisure and recreation [1,2]. However, because these parks embody such diverse values, their ecological balance is increasingly challenged by intensive human interaction.

With the growth of the tourism industry in recent years, urban forest parks have experienced an increase in visitor numbers while simultaneously suffering damage due to human activities [3,4]. Among these, tourist trampling is one of the most common forms of disturbance, exerting notable impacts on both soil and vegetation [5,6]. Previous studies have demonstrated that human trampling in scenic areas alters soil compaction and porosity, thereby affecting the soil’s capacity to retain water and nutrients, as well as its role in material cycling [7]. High-intensity trampling can also modify the chemical properties of the soil [8,9]. In addition to its effects on soil structure, trampling significantly influences microbial communities [10]. Research on the impact of trampling on soil microorganisms remains inconsistent. Some studies report that trampling reduces microbial diversity and activity [11,12], while others indicate that moderate trampling may benefit microbial communities [13]. Although many studies have examined the effects of human disturbance on soil microorganisms, the impacts on microbial community structure remain elusive.

Recent research has shifted from large forest scenic areas and alpine meadows to urban parks [14,15], yet most studies on human trampling still focus primarily on soil physicochemical properties [16]. Urban parks, as public spaces closely linked to daily human activity, experience more frequent and complex trampling disturbances. Research limited to soil physicochemical properties is therefore insufficient to reflect the overall ecosystem response. Soil microorganisms play a key role in ecosystem material cycling and energy flow, and changes in their community structure can more sensitively indicate the deeper impacts of environmental disturbance [17]. However, systematic studies examining the relationship between trampling intensity and soil microbial characteristics in urban parks remain scarce.

Zhuyuwan Scenic Area, located in Yangzhou City, Jiangsu Province, China, is a representative urban forest park facing multiple problems caused by human disturbance [18]. Accordingly, the present study selects Zhuyuwan Urban Forest Park as the research site and integrates the Neutral Community Model (NCM) with null model approaches to investigate the assembly processes of soil microbial communities under different trampling intensities. The core hypothesis is that trampling-induced changes in environmental conditions directly regulate the relative importance of stochastic and deterministic processes in microbial community assembly, with this regulatory effect varying according to the degree of trampling disturbance. These findings aim to provide a theoretical basis and technical guidance for optimizing management strategies in urban forest parks, promoting soil ecosystem stability, and ensuring sustainable utilization.

## 2. Materials and Methods

### 2.1. Soil Sample Collection

**Figure 1:**
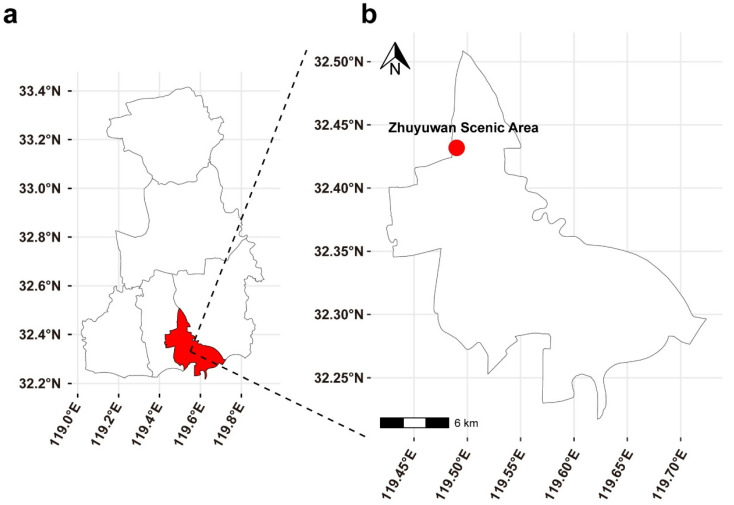
Geographical location of Zhuyuwan Scenic Area in Yangzhou City. (**a**) Longitude–latitude map of Yangzhou City. (**b**) Geographical location of Zhuyuwan Scenic Area in Yangzhou City.

**Figure 2:**
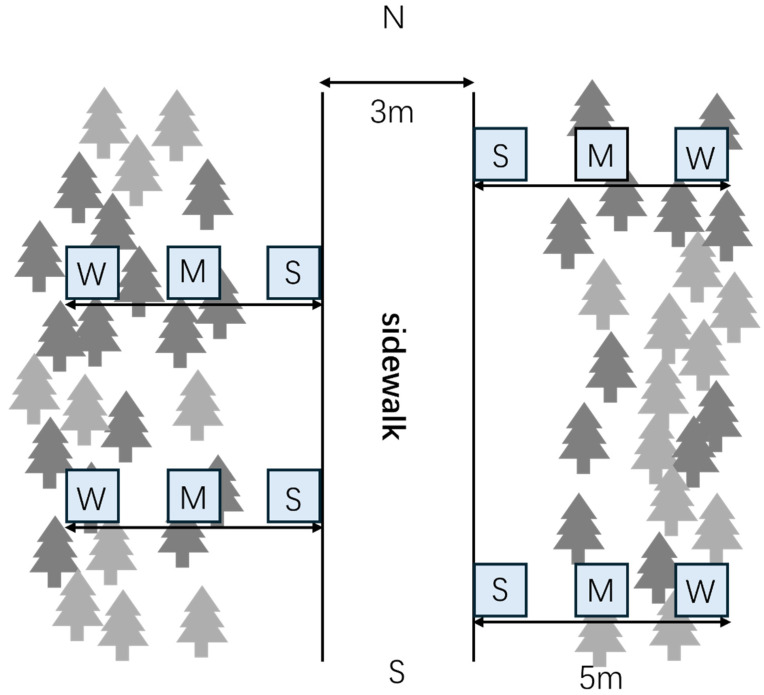
Experimental plot design. W: control, M: light trampling, S: heavy trampling.

Yangzhou, located in Jiangsu Province, China, lies in the lower reaches of the Yangtze River. The city has a developed economy, dense population, and a well-connected three-dimensional transportation network. Development zones are concentrated along the riverfront, and the city maintains a high rate of green coverage. In this study, a 3 m-wide north–south pedestrian path within the Zhuyuwan Scenic Area in Yangzhou was selected (Figure 1). Four belt transects, each measuring 5 m × 1 m and perpendicular to the path, were established on the east and west sides of the trail under similar soil and vegetation conditions. Each transect contained three 1 m × 1 m plots designated as control (W), light trampling (M), or heavy trampling (S), classified based on herb-layer damage and distance from the path edge. Heavy-trampling plots (S), which experienced intense disturbance, were centered 0.5 m from the path edge; the ground surface lacked litter, weeds, and shrubs. Light-trampling plots (M), with moderate disturbance, were centered 2.5 m from the path edge, experienced limited human activity, and supported sparse shrubs and weeds. Control plots (W), centered 4.5 m from the path edge, had dense ground cover and no evidence of trampling or strong natural recovery (Figure 2).

Soil sampling was conducted in early June 2024 using the zigzag sampling method. Soil profiles were excavated within each transect. During sampling, a soil knife was used to horizontally cut the soil along the outer side of the cutting ring to obtain intact samples. Samples that did not meet quality requirements were re-collected. Excess soil at both ends was trimmed to ensure the sample volume matched the cutting ring. In total, 36 soil samples were collected (4 transects × 9 quadrats). Each soil sample was evenly divided into three portions. One portion was used for analyzing soil physical properties, one was stored at −80 °C for bacterial 16S rRNA gene sequencing, and the remaining portion was air-dried, sieved, and used for analyzing soil chemical properties.

### 2.2. Determination of Soil Physicochemical Properties

Following the “Determination of Water-Physical Properties of Forest Soils” (LY/T 1215-1999 [19]), the ring-knife method was used to measure soil bulk density (SD), maximum water-holding capacity (MWHC), capillary water-holding capacity (CWHC), minimum water-holding capacity (MinWHC), capillary porosity (CP), and total porosity (TPor). Soil chemical properties were determined as follows: soil organic matter (SOM) was measured by the potassium dichromate volumetric method [20]; total nitrogen (TN) was digested with sulfuric acid and determined by the Kjeldahl method [21]; alkali-hydrolyzable nitrogen (HN) was determined by the alkali diffusion method [21]; available phosphorus (AP) was extracted with NaHCO_3_/NaF–HCl and measured by the Mo–Sb anticolorimetric method; available potassium (AK) was extracted with ammonium acetate and measured by flame photometry.

### 2.3. Determination of Soil Microorganisms

Total DNA was extracted from 0.5 g of soil using the FastDNA^®^ Spin Kit for Soil (MP Biomedicals, Santa Ana, CA, USA). The extracted DNA was checked on 1% agarose gel, and its concentration and purity were determined using a NanoDrop 2000 UV-Vis spectrophotometer (Thermo Scientific, Wilmington, NC, USA). The bacterial 16S V3–V4 variable region was amplified with primers 338F (5′-ACTCCTACGGGAGGCAGCAG-3′) and 806R (5′-GGACTACHVGGGTWTCTAAT-3′) [22,23]. The fungal ITS1 region was amplified using primers ITS5 (5′-GGAAGTAAAAGTCGTAACAAGG-3′) and ITS2 (5′-GCTGCGTTCTTCATCGATGC-3′) [24,25].

PCR amplification was performed using TransGen AP221-02: TransStart Fastpfu DNA Polymerase, with the ABI GeneAmp^®^ 9700as (Thermo Fisher, Shanghai, China) the PCR instrument. All samples were analyzed under standardized experimental conditions, and each sample was assayed in triplicate. The PCR products from the same sample were pooled and analyzed via 2% agarose gel electrophoresis. The products were recovered through gel extraction using the AxyPrep DNA Gel Recovery Kit (AXYGEN, Union City, CA, USA) and eluted in Tris-HCl. Subsequently, the recovered PCR products were again subjected to 2% agarose gel electrophoresis, and quantification was performed using the QuantiFluor™-ST Blue Fluorescence Quantitation System (Promega Corporation, Madison, WI, USA) based on the initial electrophoresis results. Illumina adapter sequences were attached to the ends of the target regions by PCR with the TruSeq™ DNA Sample Prep Kit. Following denaturation and annealing, the other end of the DNA fragment on the chip randomly hybridized with a neighboring primer, and paired-end (PE) reads were generated via Illumina sequencing.

### 2.4. Microbial Community Assembly Model

Research methods for microbial community assembly primarily include two types of models: the Neutral Community Model (NCM) and Null Models. The NCM evaluates the degree to which microbial community assembly is influenced by stochastic processes by estimating the relationship between microbial occurrence frequency and relative abundance. Phylogenetic null models quantify the deviation between observed phylogenetic distances and those expected under random assembly. A larger deviation indicates a stronger influence of deterministic processes, whereas a smaller deviation suggests a greater role for stochastic processes. In this study, neutral community models were constructed using R (v.4.3.2; R Foundation for Statistical Computing, Vienna, Austria) to assess the contribution of stochastic processes to microbial community assembly under different trampling intensities [26]. To further evaluate the relative contributions of stochastic and deterministic processes, the “picante” package (version 1.8.2) in R was used to build null models for deeper analysis of microbial community assembly mechanisms [27,28].

### 2.5. Statistical Analysis

A one-way analysis of variance (ANOVA) was performed on soil physical and chemical properties under different trampling intensities using basic statistical functions in R. The least significant difference (LSD) method, implemented in the “agricolae” package (version 1.3-7), was used to assess the significance of differences [29]. Cluster analysis of microbial data was conducted using the “pheatmap” package (version 1.0.13) [30], and correlations among variables were analyzed with the “corrplot” package (version 0.92) in R [31]. The significance of correlation coefficients was evaluated by calculating *p*-values. All final results were visualized using the “ggplot2” package (version 4.0.0) [32].

## 3. Results

Soil physicochemical properties are key indicators of soil quality. Compared with the control, MWHC, CWHC and MinWHC under light and heavy trampling were higher, reaching their maximum values under light trampling. The opposite trend was observed for SD, which was highest in the control and lowest under light trampling. CP and TPor increased progressively with trampling intensity (Table 1). SOM, TN, and HN decreased with increasing trampling intensity, whereas AP under both light and heavy trampling exceeded that of the control. AK reached its lowest value under heavy trampling and its highest under light trampling (Table 2).

**Figure 3:**
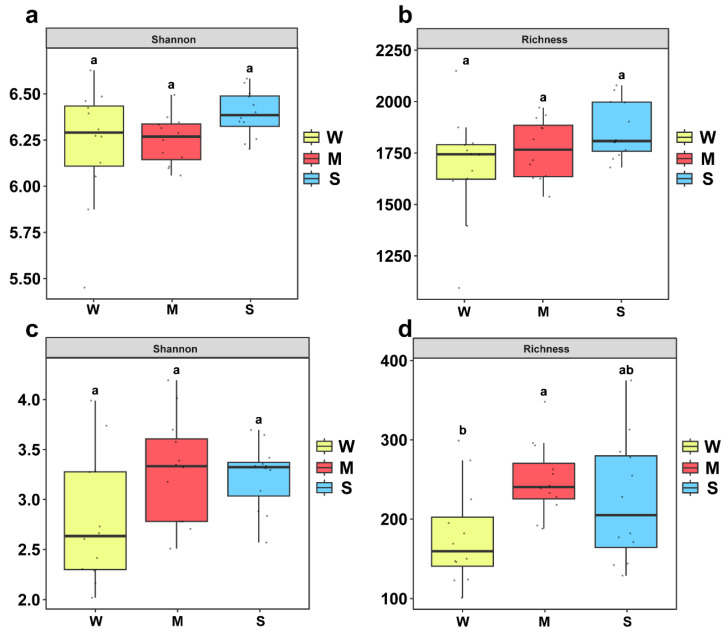
α-diversity of microbial communities under different trampling intensities. (**a**) Bacterial Shannon index; (**b**) Bacterial Richness; (**c**) Fungal Shannon index; (**d**) Fungal Richness. Lowercase letters indicate significant differences among trampling intensities (*p* < 0.05).

An α-diversity analysis of soil microorganisms under different trampling intensities showed that the bacterial Shannon index and richness were highest under heavy trampling. The bacterial Shannon index under light trampling was lower than that of the control, whereas bacterial richness under light trampling was higher than that of the control (Figure 3a,b). The fungal Shannon indices under both light and heavy trampling were higher than those of the control. Specifically, fungal richness under light trampling was the highest and significantly exceeded that of the control, while the fungal richness under heavy trampling was also higher than that of the control (Figure 3c,d).

**Figure 4:**
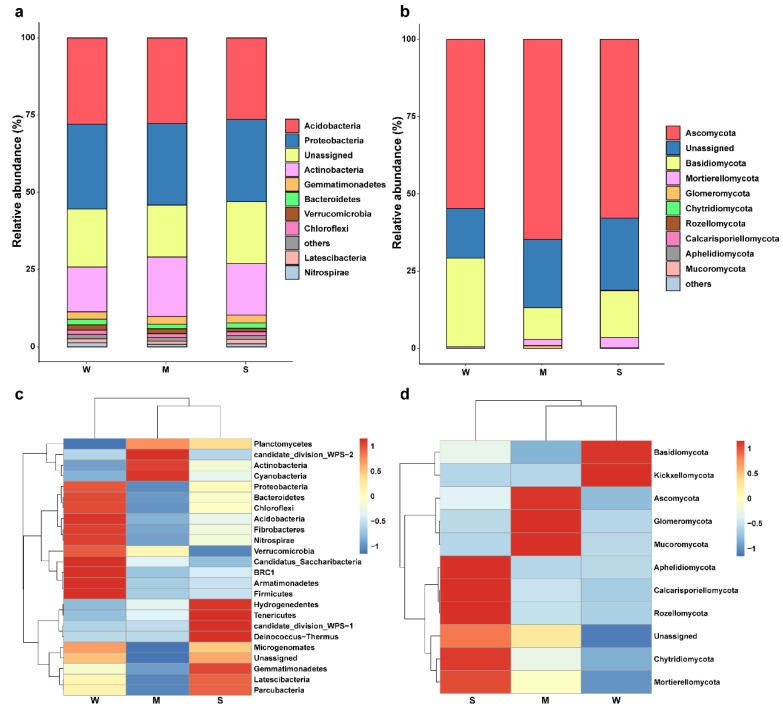
Relative abundance and cluster analysis of microbial communities at the phylum level under different trampling intensities. (**a**) Bacterial relative abundance; (**b**) Fungal relative abundance; (**c**) Bacterial cluster analysis; (**d**) Fungal cluster analysis.

The bacterial community composition under the three trampling intensities was dominated by *Acidobacteria*, *Proteobacteria*, and *Actinobacteria*. The relative abundances of *Acidobacteria* and *Proteobacteria* were similar, both ranging from 26% to 29%. In contrast, *Actinobacteria* showed a distinct pattern: its relative abundance was 14.51% in the control group and increased to 19.21% under light trampling (Figure 4a). The fungal community was mainly composed of *Ascomycota* and *Basidiomycota*. The relative abundance of *Ascomycota* ranged from 54% to 65%, while that of Basidiomycota was 28.71% in the control group, and 10.19% and 15.18% under light and heavy trampling, respectively (Figure 4b). Cluster analysis of the soil microbial communities revealed that, for bacteria, light and heavy trampling treatments had a close clustering relationship. The dominant phyla *Acidobacteria* and *Proteobacteria* exhibited the highest abundances in the control group and the lowest under light trampling, whereas *Actinobacteria* showed the opposite trend (Figure 4c). For fungi, the control and light trampling treatments were closely clustered. The abundance of the dominant phylum *Ascomycota* was highest under light trampling and lowest in the control group. In contrast, Basidiomycota abundance was highest in the control group and lowest under light trampling (Figure 4d).

**Figure 5:**
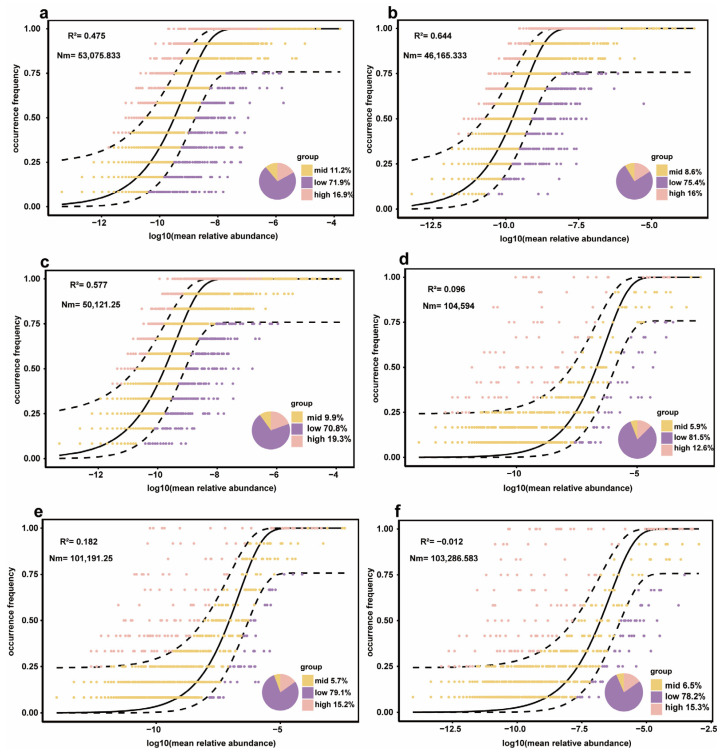
Neutral community models of microbial communities under different trampling intensities. (**a**) Bacterial community in the control group; (**b**) Bacterial community under light trampling; (**c**) Bacterial community under heavy trampling; (**d**) Fungal community in the control group; (**e**) Fungal community under mild trampling intensity; (**f**) Fungal community under heavy trampling.

A neutral community model analysis was conducted to examine microbial community assembly processes under different trampling intensities. The R^2^ values for bacterial communities under control, light, and heavy trampling were 0.475, 0.644, and 0.577, respectively, indicating that neutral processes partially explained bacterial community structure, with the strongest explanatory power under light trampling. The corresponding Nm values were 53,075.833, 46,165.333, and 50,121.25. For fungal communities, the R^2^ values were 0.096, 0.182, and −0.012, all lower than those of bacterial communities. This suggests that neutral processes had limited explanatory power for fungal community structure. The negative R^2^ under heavy trampling indicates that the neutral community model was not applicable under that condition, necessitating further analysis using a null model approach. The Nm values for fungal communities were 104,594, 101,191.25, and 103,286.583, respectively. Under corresponding trampling intensities, the Nm values of fungal communities were approximately twice those of bacterial communities (Figure 5).

**Figure 6:**
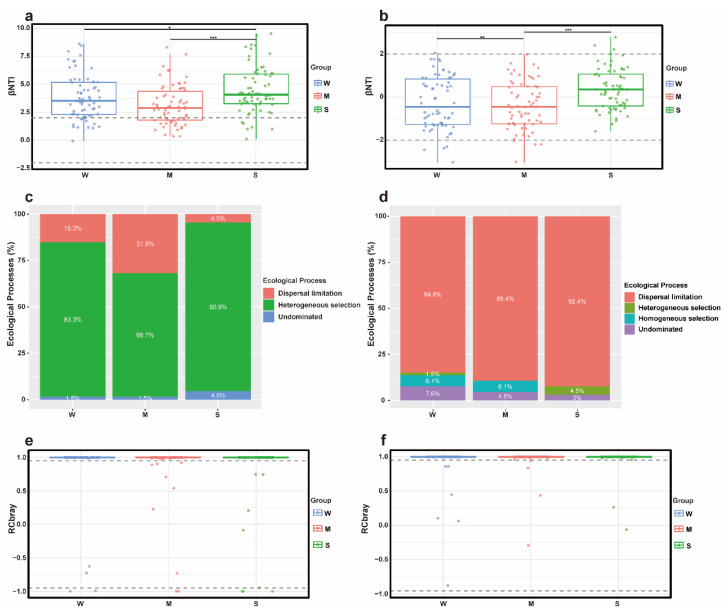
Null models of microbial communities under different trampling intensities. (**a**) βNTI of bacterial communities; (**b**) βNTI of fungal communities; (**c**) Ecological processes of bacterial communities; (**d**) Ecological processes of fungal communities; (**e**) RCbray of bacterial communities; (**f**) RCbray of fungal communities. *** Significant at the 0.001 level; ** Significant at the 0.01 level; * Significant at the 0.05 level.

To verify the results of the neutral model, a null model was constructed to further evaluate the assembly processes of soil microbial communities. The |RCbray| values of both bacterial and fungal communities were generally greater than 0.95 (Figure 6e,f). The |βNTI| values of bacterial communities across the three trampling intensities mainly ranged between 2.5 and 7.5 (Figure 6a), indicating that bacterial communities were primarily governed by deterministic processes. Significant differences in βNTI values were observed between the control and heavy trampling groups, as well as between the light and heavy trampling groups (Figure 6a). Bacterial communities across all treatments were mainly regulated by heterogeneous selection. Furthermore, compared with the control and heavy trampling groups, the light trampling group was significantly influenced by dispersal limitation (Figure 6c). The βNTI values of fungal communities ranged from −2 to 2 (Figure 6b), suggesting that fungal communities were predominantly influenced by stochastic processes. Significant differences in βNTI values were found between the light and heavy trampling groups, as well as between the light trampling and control groups. Across all treatments, fungal communities were mainly controlled by dispersal limitation. Compared with the control, the light trampling group lacked heterogeneous selection, while the heavy trampling group lacked homogeneous selection (Figure 6d).

**Figure 7:**
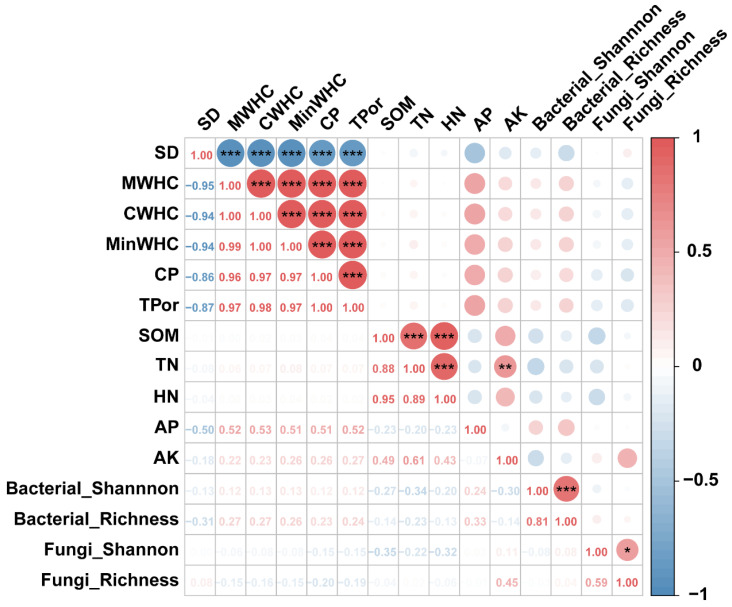
Correlation between soil physicochemical properties and microbial communities *** Significant at the 0.001 level; ** Significant at the 0.01 level; * Significant at the 0.05 level.

Correlation analysis was conducted to elucidate relationships between soil physicochemical properties and microbial communities. SD showed a highly significant negative correlation with other soil physical properties. MWHC, CWHC, MinWHC, and CP were highly significantly and positively correlated with each other. These four indicators were positively correlated with bacterial diversity but negatively correlated with fungal diversity. SOM exhibited a highly significant positive correlation with TN and HN, while TN was also highly significantly positively correlated with HN and AK. The Shannon and richness indices of microbial communities were positively correlated. Specifically, the correlation between Bacterial_Shannon and Bacterial_Richness was highly significant, while the correlation between Fungi_Shannon and Fungi_Richness was significant. Additionally, Bacterial_Shannon, Bacterial_Richness, and Fungi_Shannon were negatively correlated with SOM, TN, and HN, respectively. AP was positively correlated with all bacterial diversity indices, whereas AK showed the opposite trend. In contrast, AK was positively correlated with all fungal diversity indices (Figure 7).

## 4. Discussion

The physical and chemical properties of soil are core indicators of soil quality, and their variation is directly linked to the soil’s water and nutrient retention capacity as well as its ecological functions [33]. This study confirmed that different trampling intensities affect both the physical and chemical properties of soil. As a key indicator of soil compaction, SD strongly influences other physical parameters; effective soil water-holding capacity decreases as soil bulk density increases [34]. Soil water characteristic indicators such as MWHC, CWHC, and MinWHC all peaked under light trampling, indicating that moderate trampling can enhance the soil’s water storage and retention capacity [35]. Moreover, TPor and CP reflect the soil’s capacity for water transmission, while soils with lower total porosity often exhibit higher dry density [36]. The results of this study show that TPor and CP reached their highest values under heavy trampling, differing from the conventional view that intensified human disturbance reduces soil porosity [37,38]. This deviation may be attributed to the specific soil texture or vegetation characteristics of the study area. For soil chemical properties, SOM, TN, and HN decreased consistently with increasing trampling intensity. Previous studies have reported similar declines in soil nutrients in areas subject to human disturbance [8,39].

The microbial community functions as the “engine” of the soil ecosystem, and changes in its diversity and structure directly influence material cycling and energy flow [40,41]. Trampling intensity affected bacterial diversity to some extent but had a more pronounced effect on fungal diversity. Microbial community diversity is closely associated with soil physicochemical conditions [42,43], and trampling alters these properties, thereby influencing microbial community structure. Some studies have reported that nutrient-poor soils negatively affect bacterial communities, which differs from the findings of this study [44]. This discrepancy may result from variations in climate and vegetation among sampling areas. Indicators related to soil water content showed negative correlations with fungal community diversity, suggesting that trampling-induced changes in soil water-holding capacity may be key environmental drivers of fungal community alteration. Fungi are highly sensitive to moisture conditions and thus more susceptible to microenvironmental changes. This sensitivity may partly explain why fungal communities exhibit stronger characteristics of stochastic dispersal limitation [45]. The dominant bacterial phyla identified were *Acidobacteria*, *Proteobacteria*, and *Actinobacteria*, which together accounted for more than 70% of the total, forming the core of the soil bacterial community. Among them, *Acidobacteria* exhibited the highest relative abundance. Studies have demonstrated that *Acidobacteria* contributes to soil restoration by participating in nutrient cycling and supporting plant growth after severe disturbance [46]. This may explain why *Acidobacteria* maintained high abundance under different trampling intensities. In addition, *Proteobacteria* and *Actinobacteria* play essential roles in the global carbon cycle [47,48]. The dominant fungal phyla were *Ascomycota* and *Basidiomycota* [49]. Some studies have suggested that trampling increases the abundance of *Ascomycota* and decreases that of *Basidiomycota* [50], which contrasts with the present findings. Nonetheless, both this study and previous research indicate that *Ascomycota* remains dominant under trampling stress and can serve as an indicator taxon for assessing fungal tolerance to trampling disturbance.

When a community is subjected to disturbance, the relative contribution of stochastic processes may increase [51]. In this study, light trampling enhanced stochastic processes within the bacterial community, whereas heavy trampling reduced this effect. In contrast, as trampling intensity increased, stochastic processes played a more dominant role in fungal community assembly. Synthesizing the results of the NCM and null models, the bacterial community was found to be jointly influenced by stochastic and deterministic processes. However, some studies have reported that, although bacterial community assembly is driven by both processes, deterministic mechanisms exert slightly greater influence [52]. The NCM indicated a good fit for bacterial communities, suggesting that their assembly could be explained by neutral processes. This does not, however, exclude the role of deterministic forces. The β-nearest taxon index (βNTI) indicated that phylogenetic turnover significantly deviated from random expectations, implying that deterministic factors such as environmental filtering also play an important role in shaping bacterial community composition. Moreover, the results indicated that heterogeneous selection was the dominant deterministic process. By contrast, Mia Ridley et al. reported that homogeneous selection was the primary deterministic driver of bacterial communities [52]. This discrepancy may be attributed to the inclusion of trampling disturbance in the present study [53]. For fungal communities, the NCM fit was weak. The βNTI values of fungal communities fell within the range of −2 to 2, supporting the conclusion that stochastic processes primarily govern fungal community assembly. In general, ecological communities are jointly influenced by ecological selection, dispersal, drift, and speciation [54]. In this study, fungal community assembly was mainly affected by dispersal limitation. Although dispersal limitation alone does not directly produce spatial variations in community composition, restricted biological exchange among local communities may enhance compositional differentiation through random fluctuations in local population sizes [55].

This study examined the effects of varying trampling intensities on soil physicochemical properties, microbial community structure, and diversity, as well as their interrelationships. The findings elucidate the response mechanisms of soil ecosystems under human trampling disturbance and provide a scientific foundation for the restoration and management of degraded urban soils. Despite its valuable contributions, this study has certain limitations. First, this study did not consider seasonal variations, and fluctuations in temperature and precipitation may influence the persistence and magnitude of trampling effects. Second, functional gene expression in microbial communities was not analyzed; incorporating metagenomic sequencing is necessary to clarify the specific impacts of trampling intensity on microbial carbon and nitrogen cycling functions. Future research should therefore explore the interaction among trampling intensity, seasonal variation, and microbial functional genes to provide more precise mechanistic insights for soil ecological restoration. This study advances understanding of the impacts of human activity on urban forest soil ecosystems and offers a theoretical basis for ecological management and sustainable use of urban green spaces. Future research will expand within a “trampling–season–functional gene” framework. By integrating metagenomics, it will investigate the dynamic expression of microbial genes involved in carbon, nitrogen, and phosphorus cycling under coupled temperature and precipitation regimes. In addition, through 13C/15N labeling and microcosm experiments, it will quantify the contributions of microbial communities under different trampling intensities to organic matter decomposition and greenhouse gas fluxes. Revealing the mechanisms by which trampling influences microbial community assembly provides essential theoretical and technical guidance for the scientific management and sustainable utilization of urban forest park ecosystems.

## 5. Conclusions

This study, conducted in Yangzhou Urban Forest Park, explored the effects of anthropogenic trampling on soil physicochemical properties, microbial community structure, diversity, and assembly mechanisms. Light trampling significantly increased soil maximum water-holding capacity, capillary water content, and fungal richness, whereas heavy trampling markedly reduced soil organic matter, total nitrogen, and alkali-hydrolyzable nitrogen while intensifying soil compaction. Bacterial communities exhibited stronger stochastic processes under light trampling but were predominantly governed by deterministic processes under heavy trampling. In contrast, the stochasticity of fungal communities increased with trampling intensity. Neutral and null model analyses together indicated that bacterial communities were mainly shaped by heterogeneous selection, whereas fungal communities were primarily influenced by dispersal limitation. These findings clarify the ecological mechanisms through which trampling disturbance regulates microbial community assembly via environmental filtering and identify specific microbial taxa as potential indicators of ecosystem disturbance. The results provide a scientific basis and technical guidance for the ecological management and sustainable utilization of urban forest soil ecosystems.

## Figures and Tables

**Table 1:** Soil physical properties under different trampling intensities.

Trample Intensity	SD (g/cm^3^)	MWHC (g/kg)	CWHC (g/kg)	MinWHC (g/kg)	CP (%)	TPor (%)
W	1.39 ± 0.31 a	465.67 ± 309.72 a	455.11 ± 307.66 a	414.86 ± 298.04 a	54.68 ± 26.05 a	56.08 ± 25.92 a
M	1.20 ± 0.22 a	537.10 ± 253.00 a	512.33 ± 234.10 a	465.37 ± 230.16 a	56.74 ± 14.55 a	59.23 ± 15.16 a
S	1.32 ± 0.22 a	482.79 ± 200.12 a	470.72 ± 200.37 a	422.77 ± 190.67 a	58.33 ± 15.85 a	59.89 ± 15.57 a

SD: soil bulk density; MWHC: maximum water-holding capacity; CWHC: capillary water-holding capacity; MinWHC: minimum water-holding capacity; CP: capillary porosity; TPor: total porosity. Values are expressed as mean ± SD (*p* < 0.05). Lowercase letters indicate significant differences among trampling intensities (*p* < 0.05).

**Table 2:** Soil chemical properties under different trampling intensities.

Trample Intensity	SOM (g/kg)	TN (g/kg)	HN (mg/kg)	AP (mg/kg)	AK (mg/kg)
W	15.61 ± 10.46 a	1.59 ± 0.54 a	66.19 ± 37.62 a	10.12 ± 7.15 a	167.34 ± 58.63 a
M	15.09 ± 6.22 a	1.57 ± 0.32 a	64.41 ± 25.44 a	12.79 ± 15.81 a	181.92 ± 31.96 a
S	11.93 ± 6.01 a	1.36 ± 0.42 a	50.43 ± 22.60 a	12.53 ± 5.53 a	163.61 ± 76.82 a

SOM: soil organic matter; TN: total nitrogen; HN: alkali-hydrolyzable nitrogen; AP: available phosphorus; AK: available potassium. Values are expressed as mean ± SD (*p* < 0.05). Lowercase letters indicate significant differences among trampling intensities (*p* < 0.05).

## Data Availability

The data presented in this study are available from the corresponding author upon reasonable request due to privacy restrictions.
